# NUF2 is associated with cancer stem cell characteristics and a potential drug target for prostate cancer

**DOI:** 10.3389/fmolb.2024.1481375

**Published:** 2024-12-05

**Authors:** Dongxu Zhang, Pu Liang, Qi Wang, Bowen Xia, Liqian Yu, Xiaopeng Hu

**Affiliations:** ^1^ Department of Urology, Beijing Chaoyang Hospital, Capital Medical University, Beijing, China; ^2^ Institute of Urology, Capital Medical University, Beijing, China; ^3^ Beijing Key Laboratory of Emerging Infectious Diseases, Institute of Infectious Diseases, Beijing Ditan Hospital, Capital Medical University, Beijing, China; ^4^ Beijing Institute of Infectious Diseases, Beijing, China; ^5^ National Center for Infectious Diseases, Beijing Ditan Hospital, Capital Medical University, Beijing, China; ^6^ National Key Laboratory of Intelligent Tracking and Forecasting for Infectious Diseases, Beijing, China; ^7^ Qingdao University Medical College, Qingdao, China

**Keywords:** prostate cancer, cancer cell stemness, WGCNA, mRNAsi, biomarker

## Abstract

**Background:**

Cancer stem cells are characterized by self-renewal, clonal tumor initiation capacity, and treatment resistance, which play essential roles in the tumor progression of prostate cancer (PCa). In this study, we aim to explore the features of cancer stemness and characterize the expression of stem cell-related genes for PCa.

**Methods:**

We downloaded RNA-seq data and related clinical information from The Cancer Genome Atlas (TCGA) database. The mRNA stemness index (mRNAsi) was analyzed for various clinical features, overall survival (OS), and disease-free survival (DFS), and a weighted gene co-expression network analysis (WGCNA) was performed to identify crucial gene modules and key genes, which may play a role in CSCs. The key gene functions were verified using multiple databases, including the TCGA and Gene Expression Omnibus database (GEO). Next, we explored the potential function of the modules and genes obtained using WGCNA using an enrichment analysis. Finally, we performed *in vitro* experiments for further verification.

**Results:**

We found that mRNAsi were higher in PCa tissues than in normal tissues, and the mRNAsi were closely related to the clinical characteristics of PCa. A total of 16 key genes associated with the mRNAsi scores were identified by WGCNA analysis, including NCAPG, NEK2, DLGAP5, CENPA, CENPF, TPX2, GTSE1, KIF4A, NEIL3, CDC25C, UBE2C, CDCA5, MELK, SKA3, NUF2, and BIRC5. These genes were explicitly highly expressed in PCa across TCGA cancers and were validated in 3 independent GEO PCa datasets. The functional annotations of the key genes were linked with the cell proliferation processes. NUF2 may be a potential biomarker for PCa. *In vitro* experiments showed that knockdown NUF2 reduced the proliferation and migration of PCa cells.

**Conclusion:**

The 16 key genes identified in this study significantly correlate with PCa stem cell characteristics and showed prognosis-oriented effects in PCa patients. Further, the NUF2 gene may be used as a drug target for treating PCa.

## Introduction

Prostate cancer (PCa) is the second most frequently diagnosed cancer in men worldwide after lung cancer, with a high incidence and mortality rate (Ferlay et al., 2018). Although patients with clinically localized prostate cancer can benefit from radical prostatectomy (RP) that reduces the risk for tumor dissemination and cancer mortality, approximately 25% of patients will experience biochemical recurrence (BCR) after RP (Culp et al., 2020). The BCR of PCa has a high risk of metastasis and local recurrence. After initial androgen deprivation therapy, the vast majority of metastatic and recurrent PCa patients invariably progress to castration-resistant prostate cancer (CRPC) (Suzuki et al., 2019). Thus, there is an urgent need to explore the mechanism of PCa recurrence and find potential therapeutic targets for intervention.

Studies on intratumor heterogeneity have identified genomic heterogeneity among tumor cells, which can be comprehensively analyzed using single-cell DNA and RNA sequencing (Gerlinger et al., 2012). Cancer stem cells (CSCs) have a high proliferation and self-renewal potential in the tumor tissue (Batlle and Clevers, 2017), and these characteristics of CSCs are called cancer stemness. The CSCs in PCa have been shown to cause treatment resistance and tumor recurrence (Lytle et al., 2018), and thus, identification of the key regulators for cancer stemness and characterization of their functional impact may provide new perspectives into the progression and treatment resistance in PCa.

The stemness index is a precise and rigorous indicator representing tumor sub-populations at the molecular level. It is calculated using a one-class logistic regression (OCLR) machine learning algorithm to quantify cancer stemness (Malta et al., 2018). In a study by Malta et al., the transcriptomic and epigenetic features of pluripotent stem cells derived from normal tissues and differentiated progeny cells were extracted using the OCLR algorithm. Subsequently, a multi-platform analysis of the transcriptome, methylome, and transcription factor binding sites was performed to obtain two independent stemness indices: the DNA methylation-based stemness index (mDNAsi) and the mRNA expression-based stemness index (mRNAsi). Additionally, the value of cancer stemness from The Cancer Genome Atlas (TCGA) database was calculated and used to validate the two stemness indices. Thus, due to its reliability, it was possible to directly utilize the mRNAsi data from TCGA based on the study by Malta et al. (2018). The weighted gene co-expression network analysis (WGCNA) is a bioinformatic method used to classify genes into differential correlation clusters and calculate the relationship between the resulting clusters and various clinical features to select the modules and genes of interest (Ross-Adams et al., 2015). In this study, we aimed to identify modules of correlated differentially expressed genes (DEGs) between PCa tissues and normal tissues using WGCNA, which uses weights based on mRNA expression levels. We hypothesized that the key regulators of cancer stemness in PCa may be found by analyzing the mRNAsi modules that show significant association in a correlation analysis.

In the current study, WGCNA was used to identify stemness-related modules and 16 key genes, and we verified these genes using data from multiple databases and a multi-omics analysis. Our results provided new insights into the role of specific cancer stem cell-related genes in PCa. Moreover, we found a significant reverse correlation between the identified key gene NUF2 expression and disease-free survival (DFS). We further validated NUF2 expression in PCa tissues by immunohistochemical (IHC) staining. We also carried out a series of *in vitro* experiments to identify the role of NUF2 in PCa progression.

## Methods

### Data source

Gene expression data using the Fragments Per Kilobase of Transcript Per Million Fragments (FPKM) for PCa patients were downloaded from the TCGA database (https://portal.gdc.cancer.gov/). The mRNAsi data of PCa patients from the TCGA database was obtained from the supplement of the research article by Malta et al. (2018). The RNA-seq data, gene expression microarray files, protein levels from immunohistochemistry, and the corresponding clinical information for normal prostate and PCa tissues in the validation cohort were searched and downloaded from the Gene Expression Omnibus (GEO) database (https://www.ncbi.nlm.nih.gov/gds/) including GSE55945, GSE3325, and GSE71016.

### Evaluation of the clinical significance of mRNAsi

The FPKM and clinical data of 487 patients with PCa obtained from the TCGA database were analyzed. Next, we evaluated the significance of differences between the mRNAsi for the 499 PCa samples and 52 adjacent standard samples using the Wilcoxon signed-rank test using the beeswarm 0.2.3 R package. The correlations between the tumor stage (T stage), nodal stage (N stage), Gleason score, and mRNAsi were calculated *via* the Wilcoxon signed-rank test for the TCGA cohort using GraphPad Prism 8.0 software (GraphPad Software, San Diego, California, United States). To examine the prognostic value of the mRNAsi to predict DFS in PCa patients, we allocated the PCa patients into two groups according to the cut-off value of the mRNAsi score as calculated by X-tile. Kaplan–Meier (K–M) survival curves were analyzed using the survminer 0.4.3 R package. The gene mutation counts between the different groups were analyzed.

### Screening of DEGs

Data for 499 tissue samples from 487 patients with PCa and 52 normal tissue samples in the TCGA cohort were subjected to DEGs analysis. The DEGs between PCa samples and normal samples were identified by using the limma 3.42.0 R package *via* the Wilcoxon signed-rank test (|log2-fold change (FC)| > 1.0 and FDR <0.05) (Ritchie et al., 2015).

### WGCNA and identification of key modules and genes

A co-expression network showing DEGs was made using the WCGNA 1.68 R package (Ross-Adams et al., 2015). We first deleted the mRNA outliers in the FPKM data to do this. An adjacency matrix (AM) and a topological overlap matrix (TOM) were prepared using the gradient method based on the power values (ranging from 1 to 20). We obtained an optimal power value and constructed a scale-free topology network using a correlation value of 0.91 when comparing the average degree of connectivity (k) and p (k). The network connectivity of genes was measured using a TOM transformed from an AM (Botía et al., 2017). Modules were calculated using a divided cluster tree (Langfelder and Horvath, 2008b). We calculated the correlation between cancer stemness indices and each module eigengene to identify the most significant module. The essential modules with the highest correlation genes were selected for further analysis. We evaluated the gene significance (GS) and module membership (MM) of genes in the key modules using the correlation coefficient thresholds MM > 0.8 and GS > 0.5.

### Validation of key genes expression

To verify the relationship between the expression of key genes and the characteristics of PCa, the differential expression pattern of the key genes between PCa and normal prostate samples was validated using data from the GSE55945, GSE3325, and GSE71016 cohort *via* the ggpubr 0.2.4 R package (Ross-Adams et al., 2015).

### Relationships and interactions among hub genes

A gene expression heatmap from key genes was generated using the R package heatmap. The strength of the relationship between the expression of key genes in PCa was calculated using the Pearson correlation coefficient *via* the corrplot 0.84 R package. Networks of protein interactions were visualized with the help of the STRING database (https://www.string-db.org) (Szklarczyk et al., 2015).

### Functional enrichment analysis

To investigate the biological functions of genes in the modules, we used the Gene Ontology (GO) and Kyoto Encyclopedia of Genes and Genomes (KEGG) pathway enrichment analysis with the clusterProfiler 3.14.3 R package (Yu et al., 2012). Subsequently, the functional annotation of key genes was analyzed using Metascape (https://metascape.org/) (Zhou et al., 2019).

### Bioinformatic analysis of NUF2 in PCa

The paired and unpaired differential analysis of NUF2 expression in PCa and normal tissues was determined with the R packages “limma” and “beeswarm”. The correlation analysis of NUF2 expression and disease-free survival (DFS) was performed by R package “survival”. The Wilcox and Kruskal tests evaluated the differences in NUF2 expression at different clinical characteristics in PCa. The ROC curves were performed by “timeROC,” “survminer,” and “survival” R packages to analyze the correlation between NUF2 expression and DFS outcomes of PCa patients. The Cox regression model was used for univariate and multivariate survival analyses.

### Cell culture and transfection

The human prostate cancer cell lines PC-3 and 22RV1 were received from the Chinese Academy of Sciences Cell Bank (Shanghai, China). Cell lines were cultured according to their instruction. NUF2 and negative control (NC) siRNA were purchased from Generaybio Co., Ltd. The siRNA sequences were as follows: negative control: 5′- UUCUCCGAACGUGUCACGUTT-3′ sense and 5′- ACGUGACACGUUCGGAGAATT-3′ antisense; si-NUF2: 5′- GGAAGUGCAGUUAUAUCAATT-3′ sense and 5′- UUGAUAUAACUGCACUUCCTT-3′ antisense.

### Quantitative reverse Transcription-PCR (qRT-PCR)

Total RNA was isolated using the Total RNA Extraction Kit (Dakewe, China) and was reverse transcribed into cDNA with the PrimeScript™ RT Reagent Kit (Perfect Real Time) (TaKaRa, Japan). Next, qRT-PCR was performed using the SYBR Green PCR kit (Vazyme, China). The qRT-PCR experiments were done in triplicate. The sequences are as follows: NUF2: 5′-GCCAGACAAGAAGTGGTGGA and 3′- TTGGTCCTCCAAGTTCAGGC; β-actin: 5′- CTTCGCGGGCGACGAT and 3′- ATAGGAATCCTTCTGACCCATGC.

### Western blotting (WB)

Total proteins were extracted using RIPA buffer containing protease and phosphatase inhibitors. Protein concentration was determined by the BCA kit (Thermo Scientific, United States). Denatured proteins were fractionated by sodium dodecyl sulfate (SDS)–polyacrylamide gel electrophoresis and transferred to polyvinylidene difluoride (PVDF) membranes. Following blocking with 5% nonfat dry milk, primary and secondary antibodies were applied, and the blots were exposed using an enhanced chemiluminescence kit (Merck, Germany). The antibodies used for WB were listed below: NUF2, Abcam, United States; GAPDH, Abcam, United States. The concentration for the primary anti-NUF2 antibody was at 1:2,500 dilution, and the anti-GAPDH antibody was at 1:1,000.

### Tissue array and IHC

For the IHC of PCa patients’ tissue arrays, a tissue array chip was purchased from Shanghai Outdo Biotech Co., Ltd. (Shanghai, China). It included 30 paired tumors and adjacent normal prostate tissue samples. IHC staining was performed on paraffin-embedded sections. The samples were dewaxed and rehydrated using xylene and then passed through multiple gradient ethanol concentrations. A 3% hydrogen peroxide solution was used to block endogenous peroxidase activity, followed by further blocking with 5% bovine serum albumin. The samples were incubated with the anti-NUF2 primary antibody (Abcam, United States) at 4°C overnight. IHC staining was performed based on the manufacturer’s instructions.

Two pathologists independently reviewed and scored the IHC staining. The staining intensity was scored as follows: no staining (score = 0), weak (score = 1), moderate (score = 2), and strong (score = 3). The percentage of positively stained cells was scored as 0 (<1%), 1 (1%–25%), 2 (26%–50%), 3 (51%–75%), and 4 (76%–100%). We multiplied the scores above to obtain a final IHC score ranging from 0 to 12.

### Cell counting Kit-8 (CCK-8) experiment

Cells were seeded in a 96-well plate at a density of 5 × 10^3^ cells per well with three replicates. The manufacturer’s (MedChemExpress, China) recommended protocol was followed, and the absorbance was measured at 450 nm for 3 days.

### 5-Ethynyl-2′-deoxyuridine (EdU) experiment

The cell proliferation assay was performed using an EdU kit (Biyuntian, China). The operation procedure was carried out according to the manufacturer’s instructions. Samples were then examined by flow cytometric analysis.

### Colony formation experiment

The cells of 1 × 10^3^ were seeded in 6-cm plates and incubated at 37°C, 5% CO_2_, and 95% humidity. After 7 days, cell colonies were fixed in 4% polyformaldehyde and stained with 0.1% crystal violet.

### Wound healing experiment

The cells in the logarithmic growth phase were seeded in six-well plates, grown to 80%–90% confluency, and transfected with siRNA. After 48 h, a 10 μ L pipette tip was used to make a scratch, and then the serum-free medium was replaced. The distances of wound healing were observed, and images were collected at 0 h, 12 h, and 24 h after injury.

### Transwell experiment

Cells of 5 × 10^3^ were seeded into the serum-free medium in the upper chamber, and the lower chamber of the Transwell was added with a complete medium. After 48h, the cells were fixed with 4% paraformaldehyde and stained with 0.05% crystal violet staining solution. Cells at the bottom of the chamber were counted.

## Results

### The relationship between mRNAsi to clinical features of PCa

**FIGURE 1:**
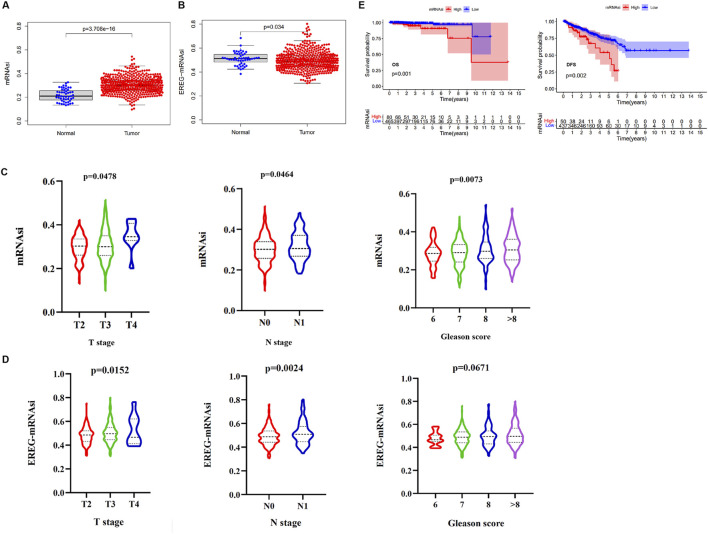
The relationship between the mRNAsi and clinical variables or prognosis. **(A)** The differences in mRNAsi between normal (52 samples) and tumor (499 samples). **(B)** The differences in EREG-mRNAsi between normal (52 samples) and tumor (499 samples). **(C)** Comparison of mRNAsi in different T stage, N stage, and Gleason scores of PCa. **(D)** Comparison of EREG-mRNAsi in different T stage, N stage, and Gleason scores of PCa. **(E)** Kaplan-Meier (K–M) curves showing the OS and DFS of PCa patients with low and high mRNAsi based on the median cutoff point.

To explore the clinical significance of mRNAsi, which can be considered as a marker for CSCs, the mRNA expression data, mRNAsi, and clinical data in the TCGA cohort from a total of 499 samples from 487 patients having PCa, and 52 samples from normal adjacent tissue were utilized. We found that both mRNAsi and epigenetically regulated mRNAsi (EREG-mRNAsi) in PCa samples were significantly higher than adjacent normal samples (Figures 1A, B). Next, the mRNAsi between PCa samples for different T stage, N stage, and Gleason score were investigated. The mRNAsi scores were significantly higher in patients with a higher T stage, N stage, and Gleason score (Figure 1C). Similarly, the EREG-mRNAsi score was significantly associated with the T and N stages but not with the Gleason scores (Figure 1D). Furthermore, we classified the patients with PCa into two groups based on the mRNAsi score. The results of the K-M survival analysis indicated that patients with high mRNAsi scores had a decreased OS and DFS time compared to those with a low score (Figure 1E). In contrast, there was no significant difference in OS and DFS between the high and low EREG-mRNAsi groups (Supplementary Figure S1). These results revealed that the mRNAsi was closely related to the clinical characteristics of PCa, which may indicate a close relationship between the CSCs and the progression of PCa.

### Verification of cancer stemness-related modules and genes

**FIGURE 2:**
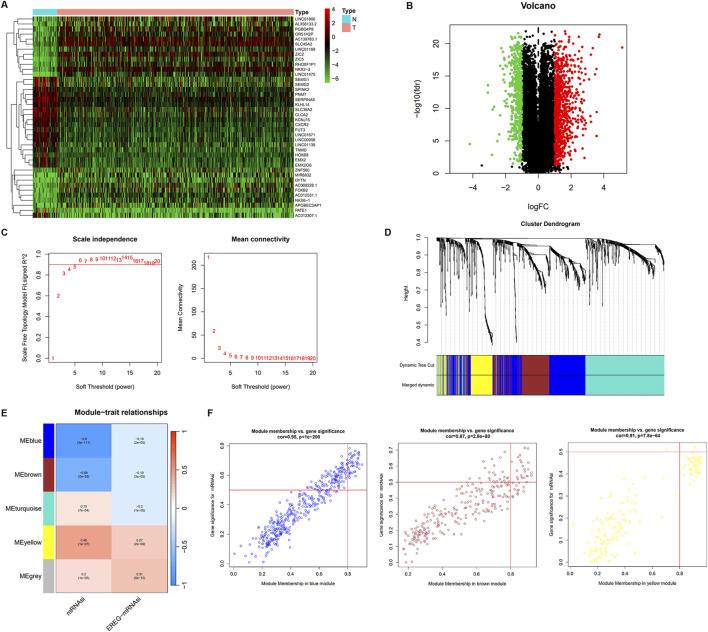
The different expression mRNA and weighted gene co-expression network of PCa. **(A)** The heatmap of different expression mRNA expression between normal and tumor samples. **(B)** The different expression mRNA between normal and PCa samples; green indicates downregulated genes, and red indicates upregulated genes. **(C)** Analysis of network topology for various soft-thresholding powers in scale independence and mean connectivity. **(D)** Identification of a co-expression module in PCa. The branches of the cluster dendrogram correspond to the five different gene modules. Each piece of the leaves on the cluster dendrogram corresponds to a gene. **(E)** Heatmap of the correlation between module eigengenes and clinical traits. The clinical traits include mRNAsi and EGER-mRNAsi. Each cell contains the corresponding correlations and P values. **(F)** Scatter plot of module eigengenes in the blue, brown, and yellow modules.

To identify the key regulators of cancer stemness in PCa, we performed a differential expression analysis to identify DEGs in a comparison between the 499 PCa samples and 52 normal samples using the limma R package. A total of 1,391 DEGs were identified, of which 895 were upregulated, and 496 were downregulated relative to genes from normal tissue (Figures 2A, B; Supplementary Table S1). Next, we constructed a scale-free co-expression network using the 1,391 DEGs *via* WGCNA using the power of β = 6 (Figure 2C). We obtained 5 modules containing DEGs as partitioned by the average linkage hierarchical clustering method. Subsequently, we merged the highly similar modules using the dynamic hybrid tree cut method (cut line = 0.22; minimum module size = 50; Figure 2D). We identified the yellow modules containing 164 genes related closely to PCa cancer stemness (Supplementary Table S2). The module-trait correlation of these modules with mRNAsi was 0.46, and EREG-mRNAsi was 0.27 (Figure 2E). On applying thresholds of MM > 0.8 and GS > 0.5, 16 key genes including NCAPG, NEK2, DLGAP5, CENPA, CENPF, TPX2, GTSE1, KIF4A, NEIL3, CDC25C, UBE2C, CDCA5, MELK, SKA3, NUF2, and BIRC5, were identified from the yellow modules (Figure 2F).

### Analysis and validation of key genes expression

**FIGURE 3:**
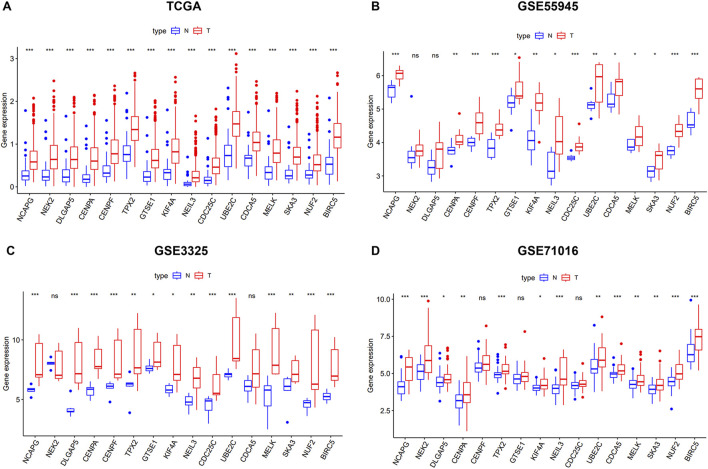
The 16 key genes were verified in the GEO database. **(A)** The difference of expression of key genes in normal and tumor of PCa in TCGA. **(B)** The difference of expression of key genes in normal and tumor of PCa in GSE55945. **(C)** The difference of expression of key genes in normal and tumor of PCa in GSE3325. **(D)** The difference of expression of key genes in normal and tumor of PCa in GSE71016.

**FIGURE 4:**
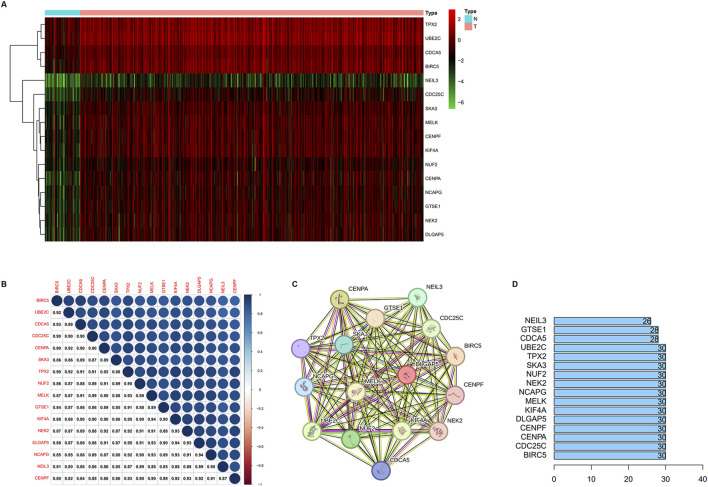
**(A)** The heatmap of representative key DEGs. **(B)** Correlation analysis between key DEGs. **(C)** The PPI network between the key genes of the yellow module. **(D)** The number of solid lines of protein in the protein-protein interaction net.

We verified the differential expression level of the key genes using TCGA and GEO databases (GSE55945, GSE3325, and GSE71016). The results showed that the key genes were significantly upregulated in the PCa samples relative to the normal prostate samples in four cohorts (Figures 3A–D). Meanwhile, we generated a heatmap using the R package “heatmap” to display the key genes between tumor and normal tissues (Figure 4A). Further, a Pearson correlation coefficient analysis between the key genes for mRNA expression revealed a strong and significant correlation between them, with KIFA4 and TPX2 having the highest correlation coefficient of 0.95 and CENPF and BIRC5 showing the lowest correlation coefficient of 0.80 (Figure 4B). We also constructed a PPI network of the 16 key genes using the STRING database, and Cytoscape software was used to visualize the PPI network (Figure 4C). We also found that there were strong connections between these genes. In the PPI network, the nodes with a high degree of connection were considered hub proteins. The NUF2, NCAPG, NEK2, DLGAP5, CENPA, CENPF, TPX2, KIF4A, CDC25C, UBE2C, MELK, SKA3, and BIRC5 had the most edges in the PPI network (Figure 4D).

### Functional enrichment analyses of modules and key genes

**FIGURE 5:**
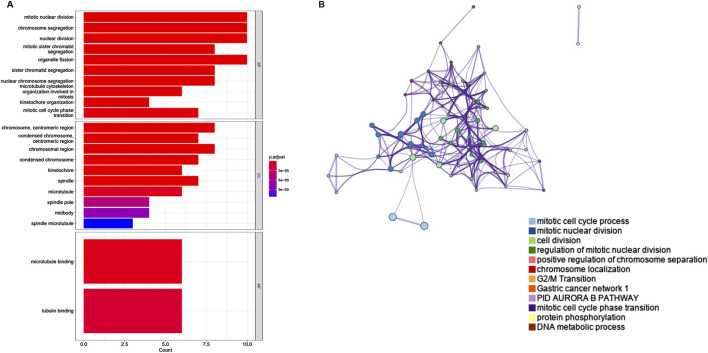
Enrichment analyses of the significant module. **(A)** GO enrichment analysis of the yellow module. **(B)** The functional enrichment analysis of key genes with Metascape.

To explore the functions associated with the yellow gene modules, we used the “clusterProfiler” for GO/KEGG enrichment, and the results showed that the main functions related to the key modules were mitotic nuclear division, chromosome segregation, nuclear division, tubulin binding, and microtubule binding, which are primarily linked to cell proliferation (Figure 5A). To identify the biological functions of the key genes, we utilized Metascape, a free online tool for gene annotation. The key genes showed significant enrichment for the mitotic cell cycle phase transition (Figure 5B).

### Confirmation of NUF2 expression and evaluation of the prognostic value of NUF2 in PCa

**FIGURE 6:**
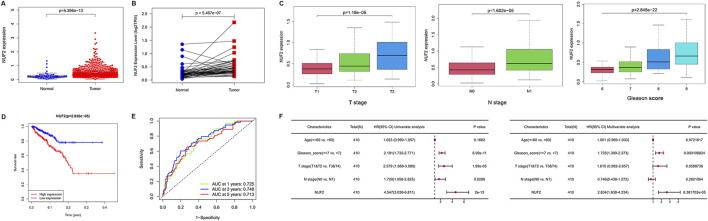
Validation of NUF2 expression in PCa and evaluation of its prognostic value. **(A, B)** Comparison of NUF2 expression in paired and non-paired groups. **(C)** Associations between NUF2 expression and different T stage, N stage, and Gleason scores in PCa patients. **(D)** Associations between NUF2 expression and the DFS in PCa patients. **(E)** ROC analysis of DFS prediction in the TCGA cohort. **(F)** Forest plot showing the results of univariate and multivariate Cox regression analysis.

The paired difference analysis and unpaired difference analysis using the TCGA database indicated that NUF2 was significantly overexpressed in PCa tissues compared with normal tissues (Figures 6A, B). In addition, we explored the correlation between NUF2 and different clinical subgroups in PCa. The results showed that elevated NUF2 expression was significantly associated with T stage, N stage, and Gleason score in PCa patients (Figure 6C). High NUF2 expression also indicated unfavorable DFS in PCa (Figure 6D), while its expression did not correlate with OS (Supplementary Figure S2). ROC curves showed a favorable predictive capacity of NUF2 expression for the 1/3/5-year DFS in the TCGA cohort (Figure 6E). Cox survival analysis showed a significant difference in prognosis between the high and low NUF2 expression groups, both univariate [Hazard ratio (HR): 4.547, 95% confidence interval (CI): 3.036–6.811, *p* < 0.001] and multivariate (HR: 2.634, 95% CI: 1.638–4.234, *p* < 0.001) (Figure 6F). NUF2 expression, age, T stage, and Gleason score were independent prognostic factors of KIRC.

### Knockdown of NUF2 expression reduced cell proliferation and migration of PCa cells

**FIGURE 7:**
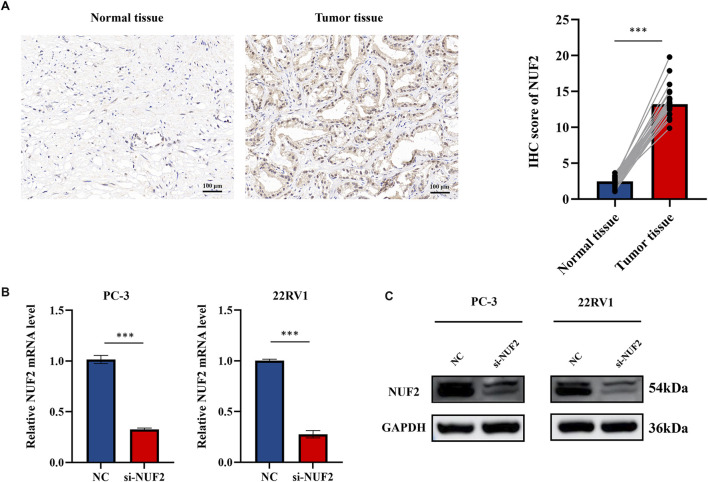
**(A)** IHC staining of NUF2 in PCa and paracancerous tissues. **(B, C)** Verification of knockdown efficiency of NUF2 in PC-3 and 22RV1 cell lines by qRT-PCR and WB (*p < 0.05, **p < 0.01, ***p < 0.001).

**FIGURE 8:**
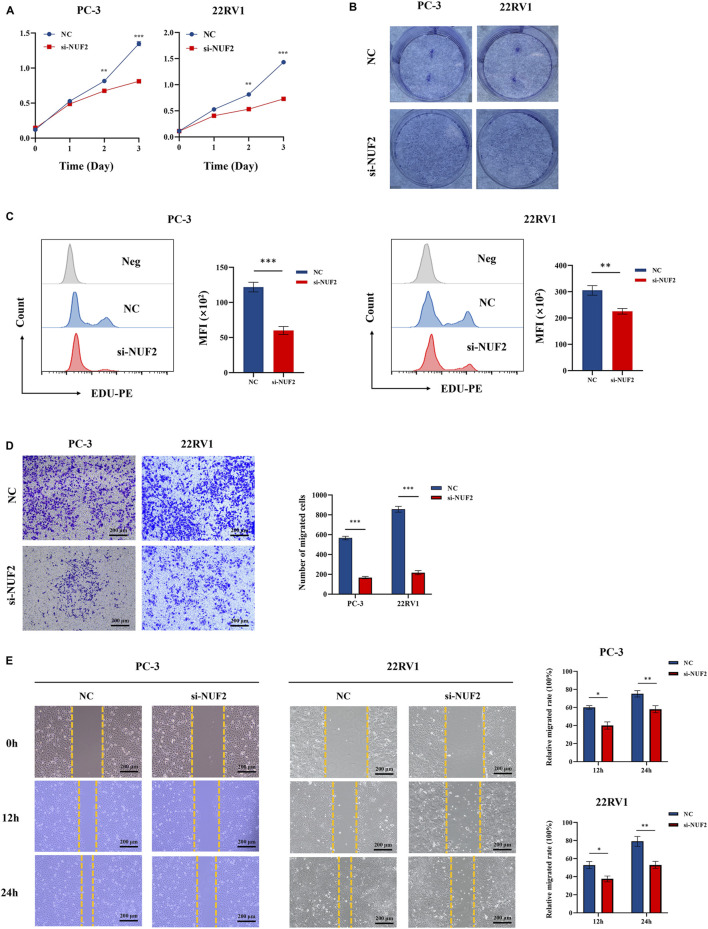
The biological functions of NUF2 in PCa. **(A–C)** Cell proliferation was assessed by CCK experiment, EdU experiment, and clone formation experiment. **(D–E)** Cell migration was assessed by transwell experiment and wound healing experiment (*p < 0.05, **p < 0.01, ***p < 0.001).

Experiments were conducted to further verify the bioinformatics above analysis results. Firstly, we detected NUF2 expression in PCa tumor tissues and their corresponding adjacent normal tissues by IHC analysis. The results indicated that NUF2 was primarily expressed in the cytoplasm of PCa cells and that the protein expression level of NUF2 in tumor tissues was significantly higher than in normal prostate tissues (Figure 7A). To probe the biological function of NUF2, the siRNA knockdown of NUF2 was performed in PC-3 and 22RV1 cells. WB analysis and qRT-PCR confirmed the efficiency of the knockdown (Figures 7B, C). CCK8 experiments showed that NUF2 knockdown significantly suppressed PC-3 and 22RV1 cell viability (Figure 8A). The capability of cell proliferation was tested using the colony formation experiment and EdU experiment. We observed that the NUF2 knockdown strongly reduced the number of colonies and proliferative capacity of PC-3 and 22RV1 cells (Figures 8B, C). In addition, we performed a transwell and wound healing experiment to verify the effect of NUF2 on the migratory ability of PCa cells. The results showed that NUF2 knockdown suppressed the function of PCa cell migration (Figures 8D, E). These results showed that NUF2 promoted the proliferation and migration of PCa cells *in vitro*.

## Discussion

PCa is a common urinary disease associated with a high recurrence rate and a poor prognosis. In recent years, a growing body of evidence has indicated that CSCs could lead to prostate cancer progression and therapeutic resistance. Thus, understanding the characteristics and seeking biomarkers for CSCs is critical for treating PCa (Abd Elmageed et al., 2014). Our study found that the mRNAsi scores increased as the T stage, N stage, and Gleason scores increased. A higher mRNAsi score was associated with shorter OS and DFS in PCa. Further, using WGCNA, we screened the 16 identified key genes based on the mRNAsi score and validated the differential expression of these genes in PCa using GEO-independent datasets. In addition, we found that key genes are strongly co-expressed and that key genes may interact with each other to influence each other’s transcript levels, and this characteristic may be used to predict the prognosis of PCa patients. We also identified multiple core cancer stemness regulatory genes, which might be of interest as drug targets in the future. Thus, using *in silico* analysis, our study identified an essential role for CSCs in PCa and identified several potential biomarkers of CSCs in PCa.

Multiple studies found in the literature have suggested that cancers of human cells are stem cell diseases, and the oncogenesis and development of tumors have a close relationship with a few subtypes of cancer cells having stem cell-like features. According to one study for PCa, the cancer stemness increased significantly with the tumor progression (Yun et al., 2016). In our research, we found that the CSC characteristics of PCa were correlated with an increasing T stage, N stage, and Gleason score, indicating that stem cell properties might promote the development of PCa. The results of the survival analysis revealed that patients with higher mRNAsi scores had shorter OS and DFS, which was similar to patients with tumors having prominent CSC characteristics and who had a poor prognosis.

Functional enrichment analysis of the yellow module showed that the module genes were primarily associated with mitotic nuclear division, chromosome segregation, and atomic division processes. Additionally, the functions of the key genes, as confirmed by the WGCNA analysis, were similar to those in the yellow module. A strong protein interaction relationship was discovered among key genes. An analysis of protein function identified NUF2, NCAPG, NEK2, DLGAP5, CENPA, CENPF, TPX2, KIF4A, CDC25C, UBE2C, MELK, SKA3, and BIRC5 as playing a central role in the protein network obtained, indicating that they may be potential drug therapeutic targets. Previous research has shown that CSCs regulate the occurrence and development of cancer through the PI3K/AKT/mTOR, Wnt/β-catenin, Notch, and Hedgehog pathways (Xia and Xu, 2015; Takebe et al., 2015). Among the key genes, NUF2, CENPA, and UBE2C were found to be involved in the PI3K/AKT/mTOR pathway (Ren et al., 2023; Zhang et al., 2021; Zhu et al., 2021). NUF2, NCAPG, NEK2, and DLGAP5 were found to be involved in the Wnt/β-catenin pathway (Li et al., 2016; Shi et al., 2022; Jeon et al., 2024; Chen et al., 2023; Wang et al., 2021). Together, these results indicate that these genes may play an essential role in the tumorigenicity of CSCs.

NUF2 is a key molecule for stabilizing spindle microtubule attachment at the metaphase of cell division, and it is a component of the NDC80 mitotic complex (Nabetani et al., 2001; DeLuca et al., 2003). Previous studies have confirmed that abnormal segregation of chromosomes during mitosis is a common cause of cancer (Cheerambathur et al., 2013; Foley and Kapoor, 2013). DeLuca et al. found that the knockdown of NUF2 in HeLa cells resulted in the inability of the kinetochores to connect with spindle microtubules, leading to abnormal chromosome division and apoptosis (DeLuca et al., 2002). Thus, it is not unexpected that dysregulation of NUF2 expression and function can promote tumor formation. Many reports have identified NUF2 as an oncogene in various cancers, including clear cell renal cell carcinoma (Lin et al., 2022), melanoma (Wang et al., 2022), and breast cancer (Lv et al., 2020). These results suggest that NUF2 plays a crucial role in cancer cells, while the precise role of NUF2 in PCa has not been clarified. Our results showed that mRNA and protein abundance of NUF2 was higher in PCa tumor tissues, and a high NUF2 expression level was associated with poorer DFS in PCa patients. Likewise, the ROC curve showed that the positive expression of NUF2 was a good predictor of DFS in PCa patients. Univariate and multivariate Cox regression analyses showed that NUF2 was an independent predictive factor for the prognosis of PCa patients. Moreover, our vitro experiments also showed that NUF2 was highly expressed in PCa tissues, and knockdown of NUF2 may significantly inhibit the proliferation and migration ability of PC-3 and 22RV1 cells.

To our knowledge, few similar studies use WGCNA combined with tumor stem cells to predict PCa-related genes. For the first time, our study leveraged high-throughput sequencing data from public databases and the WGCNA method to obtain modules in PCa that are closely related to stem cell characteristics and explored the biological role of the cancer stem cell characterization-related gene NUF2 in PCa. However, there were still certain limitations in the present study. Firstly, our study only conducted *in vitro* and lacked *in vivo* animal experiments. In future studies, we will employ further experiments for validation *in vivo*. Second, because our research data come from public databases, the quality of these data may not be guaranteed. Therefore, further extensive sample-size biological studies are needed to confirm our findings.

## Conclusion

In summary, we identified 16 key genes that may influence stem cell maintenance in PCa. NUF2 may play a critical role in developing PCa by affecting cancer stemness. Targeting the gene NUF2 may be a potential treatment strategy for retaining PCa stemness characteristics.

## Data Availability

The raw data supporting the conclusions of this article will be made available by the authors, without undue reservation.
