# Supplementary material for: NUF2 is associated with cancer stem cell characteristics and a potential drug target for prostate cancer
Source: Front Mol Biosci. 2024 Dec 5;11:1481375. doi: 10.3389/fmolb.2024.1481375 (PMC11656027; doi:10.3389/fmolb.2024.1481375)
Supplement: Supplementary file 1 [file DataSheet1.docx]

**Title**: NUF2 is Associated with Cancer Stem Cell Characteristics and are Potential Drug Targets for Prostate Cancer

**Author**: Dongxu Zhang^1,2†^, Pu Liang^4,5,6,7†^, Qi Wang^4,5,6,7†^, Bowen Xia^1,2^, Liqian Yu^3*^and Xiaopeng Hu^1,2*^

1. Department of Urology, Beijing Chaoyang Hospital, Capital Medical University, NO. 8 Gongti South Road, Beijing, China

2. Institute of Urology, Capital Medical University, Beijing, China

3. Qingdao university medical college, Qingdao, China

4. Beijing Key Laboratory of Emerging Infectious Diseases, Institute of Infectious Diseases, Beijing Ditan Hospital, Capital Medical University, Beijing 100015, China

5. Beijing Institute of Infectious Diseases, Beijing 100015, China

6. National Center for Infectious Diseases, Beijing Ditan Hospital, Capital Medical University, Beijing 100015, China

7. National Key Laboratory of Intelligent Tracking and Forecasting for Infectious Diseases, Beijing 100015, China

Dongxu Zhang, Pu Liang and Qi Wang contributed equally to this work as co-first authors.

*Correspondence

Xiaopeng Hu^1,2*^ and Liqian Yu^3*^

1. Department of Urology, Beijing Chaoyang Hospital, Capital Medical University, NO. 8 Gongti South Road, Beijing, China

2. Institute of Urology, Capital Medical University, Beijing, China

3. Qingdao university medical college

Tel: 86- 010-85231777

Fax: 86- 010-85231777

e-mail: scitongxunzuozhe@163.com, yuliqian62@hotmail.com


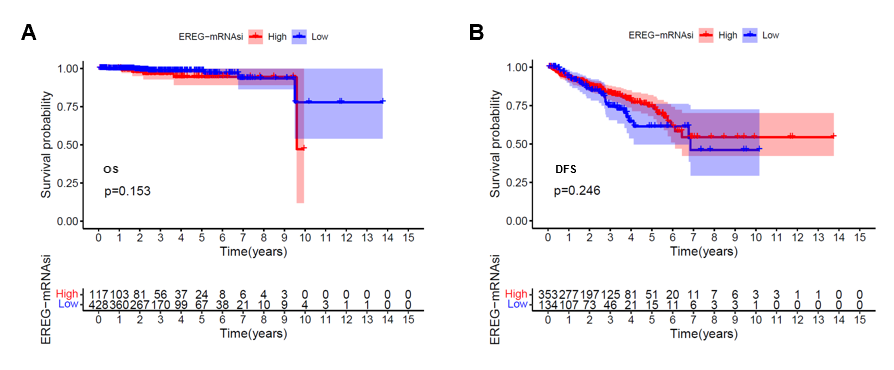


**Supplementary Figure 1.** Kaplan-Meier (K-M) curves showing the (A) OS and (B) DFS of PCa patients with low and high EREG-mRNAsi based on the median cutoff point.


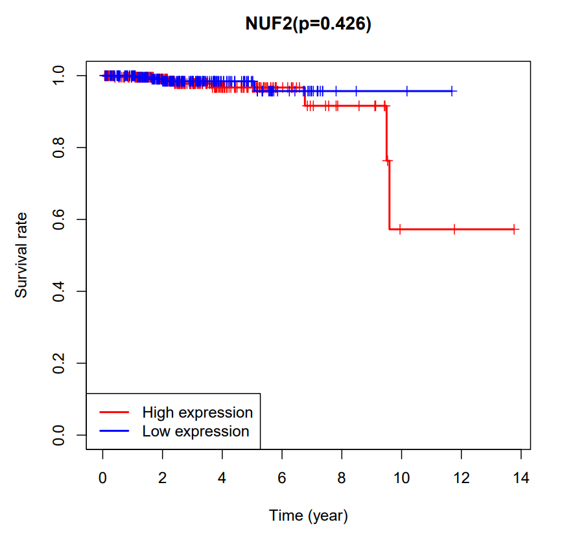


**Supplementary Figure 2.** Associations between NUF2 expression and the OS in PCa patients.


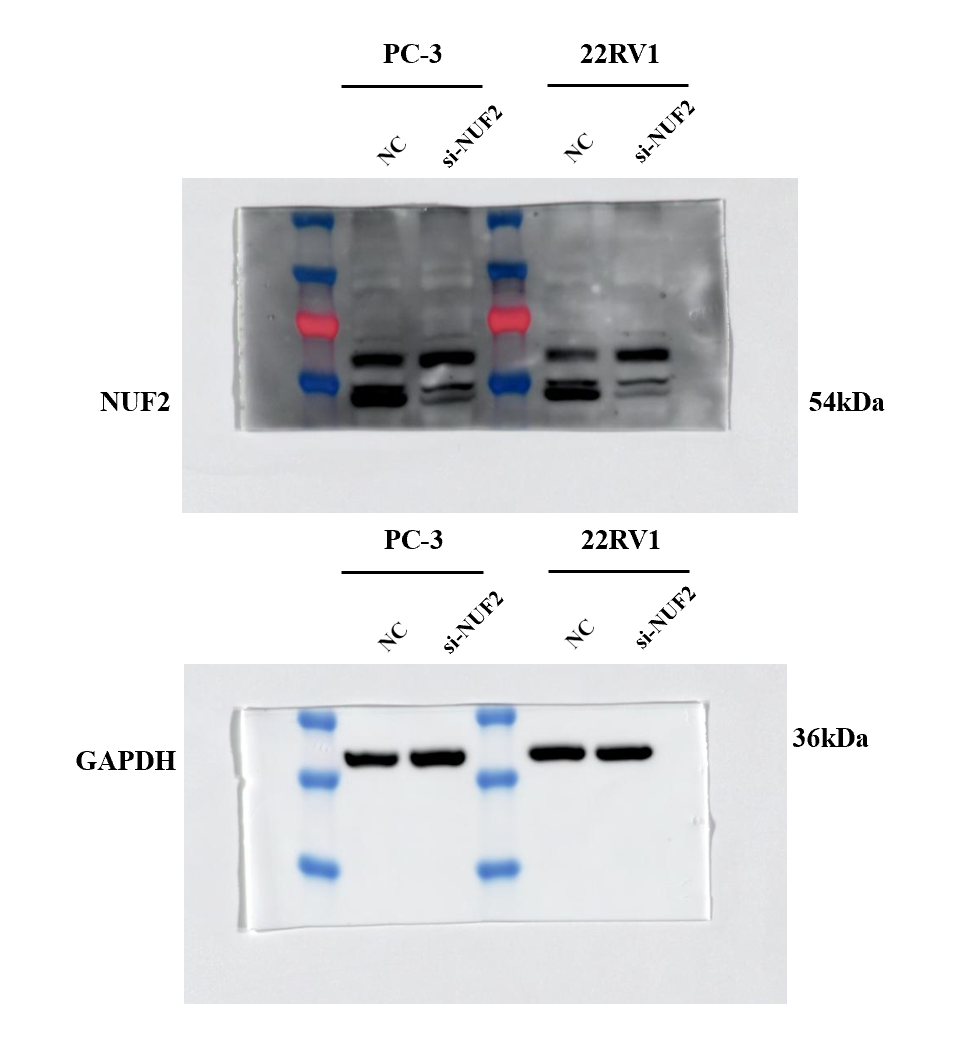


**Supplementary Figure 3.** The original western blots.
